# Modulators of Cytoskeletal Reorganization in CA1 Hippocampal Neurons Show Increased Expression in Patients at Mid-Stage Alzheimer's Disease

**DOI:** 10.1371/journal.pone.0013337

**Published:** 2010-10-13

**Authors:** Patricia F. Kao, David A. Davis, Meredith G. Banigan, Charles R. Vanderburg, Sudha Seshadri, Ivana Delalle

**Affiliations:** 1 Department of Pathology and Laboratory Medicine, Boston University School of Medicine, Boston, Massachusetts, United States of America; 2 Advanced Tissue Research Center, Harvard NeuroDiscovery Center, Massachusetts General Hospital, Charlestown, Massachusetts, United States of America; 3 Department of Neurology, Boston University School of Medicine, Boston, Massachusetts, United States of America; Mental Health Research Institute and the University of Melbourne of Victoria, Australia

## Abstract

During the progression of Alzheimer's disease (AD), hippocampal neurons undergo cytoskeletal reorganization, resulting in degenerative as well as regenerative changes. As neurofibrillary tangles form and dystrophic neurites appear, sprouting neuronal processes with growth cones emerge. Actin and tubulin are indispensable for normal neurite development and regenerative responses to injury and neurodegenerative stimuli. We have previously shown that actin capping protein beta2 subunit, Capzb2, binds tubulin and, in the presence of tau, affects microtubule polymerization necessary for neurite outgrowth and normal growth cone morphology. Accordingly, Capzb2 silencing in hippocampal neurons resulted in short, dystrophic neurites, seen in neurodegenerative diseases including AD. Here we demonstrate the statistically significant increase in the Capzb2 expression in the postmortem hippocampi in persons at mid-stage, Braak and Braak stage (BB) III-IV, non-familial AD in comparison to controls. The dynamics of Capzb2 expression in progressive AD stages cannot be attributed to reactive astrocytosis. Moreover, the increased expression of Capzb2 mRNA in CA1 pyramidal neurons in AD BB III-IV is accompanied by an increased mRNA expression of brain derived neurotrophic factor (BDNF) receptor tyrosine kinase B (TrkB), mediator of synaptic plasticity in hippocampal neurons. Thus, the up-regulation of Capzb2 and TrkB may reflect cytoskeletal reorganization and/or regenerative response occurring in hippocampal CA1 neurons at a specific stage of AD progression.

## Introduction

Genes implicated in the development of AD influence microtubule and actin filaments responsible for neuronal morphology[1]. The presence of an apolipoprotein E4 allele[2], correlates with the simplification of dendritic branching patterns in the brains of AD patients[3]. Consistent with this observation in human brains, the apolipoprotein E4 inhibits neurite outgrowth in cultured neuronal cells[4]. Interestingly, amyloid precursor protein concentrates in lamellipodia where it is proposed to play a role in growth cone motility and neurite outgrowth[5].

Upon acute neuronal injury, such as axotomy, the first critical steps that initiate regenerative response are microtubule polymerization and F-actin cytoskeleton rearrangement leading to the formation of a motile growth cone in a stable axonal segment[6]. Actin cytoskeleton regulator CP (F-actin capping protein, CapZ) is an α/β heterodimer that binds the barbed end of F-actin thus blocking the access of actin monomers to the fast growing end. Both mammalian and *Drosophila* CP subunits play a critical role in the organization and dynamics of lamellipodia and filopodia in non-neuronal cells[7]. One of the mammalian β-subunit isoforms, Capzb2, is predominantly expressed in the brain[8]. We have demonstrated that Capzb2 not only caps F-actin barbed end but also binds βIII-tubulin directly, affecting the rate and the extent of microtubule polymerization in the presence of tau[9]. Moreover, Capzb2 - βIII-tubulin interaction is indispensable for normal growth cone morphology and neurite length[9].

The interaction between CapZ and β-tubulin was uncovered in a mass spectrometry screen for altered protein-protein interactions in response to spatial learning[10]. CapZ localization in the hippocampal dendritic spines has been recently shown to undergo activity-dependent, synapse-specific regulation in a rat model of dementia[11]. BDNF is necessary for normal spatial learning[12] and reduced BDNF and TrkB mRNA levels correlate with impaired memory performance in senescent rats[13], [14]. Further, lifestyle modifications that are thought to reduce the risk of developing clinical AD, such as intake of docosahexaenoic acid (DHA) and increased exercise, appear to interact with BDNF-related synaptic plasticity[15]. Actin cytoskeleton is a well-established target for BDNF/TrkB signaling that affects not only memory formation and retention[16], [17] but also neuronal regeneration[18]. BDNF is required for normal F-actin distribution in growth cones and for axonal protrusion during regeneration in retinal ganglion cells[19].

As hyperphosphorylated tau gives rise to neurofibrillary tangles in AD, dystrophic neurites, marked by reduced length and poor branching, become apparent. In parallel, perisomatic proliferation of dendrites and sprouting of distal dystrophic neurites take place[20]. The presence of growth cone-like structures on distal ends of dystrophic neurites suggests that regenerative response accompanies degenerative cytoskeletal changes in AD[20]. These morphological changes in neurons during AD progression indicate major cytoskeletal reorganization raising the possibility that microtubules and microfilaments may represent a target for pathobiological mechanisms underlying AD. Here we report a significant increase in Capzb2 protein and mRNA levels in hippocampal CA1 pyramidal neurons at mid-stage non-familial AD (Braak and Braak, BB, stage III-IV). The up-regulation of Capzb2 at this stage is accompanied by an increase in mRNA levels of BDNF primary receptor, TrkB. BDNF/TrkB signaling modulates cell morphology and neurite length[21], [22]. Our data suggest that Capzb2, a recently established link in microfilament - microtubule assembly, together with BDNF/TrkB signaling, may play a role in cytoskeletal reorganization and possibly regenerative changes at specific stages of AD progression.

## Results

### Capzb2 protein expression shows a specific increase in the hippocampal CA1 region in AD BB stage III-IV

**Figure 1:**
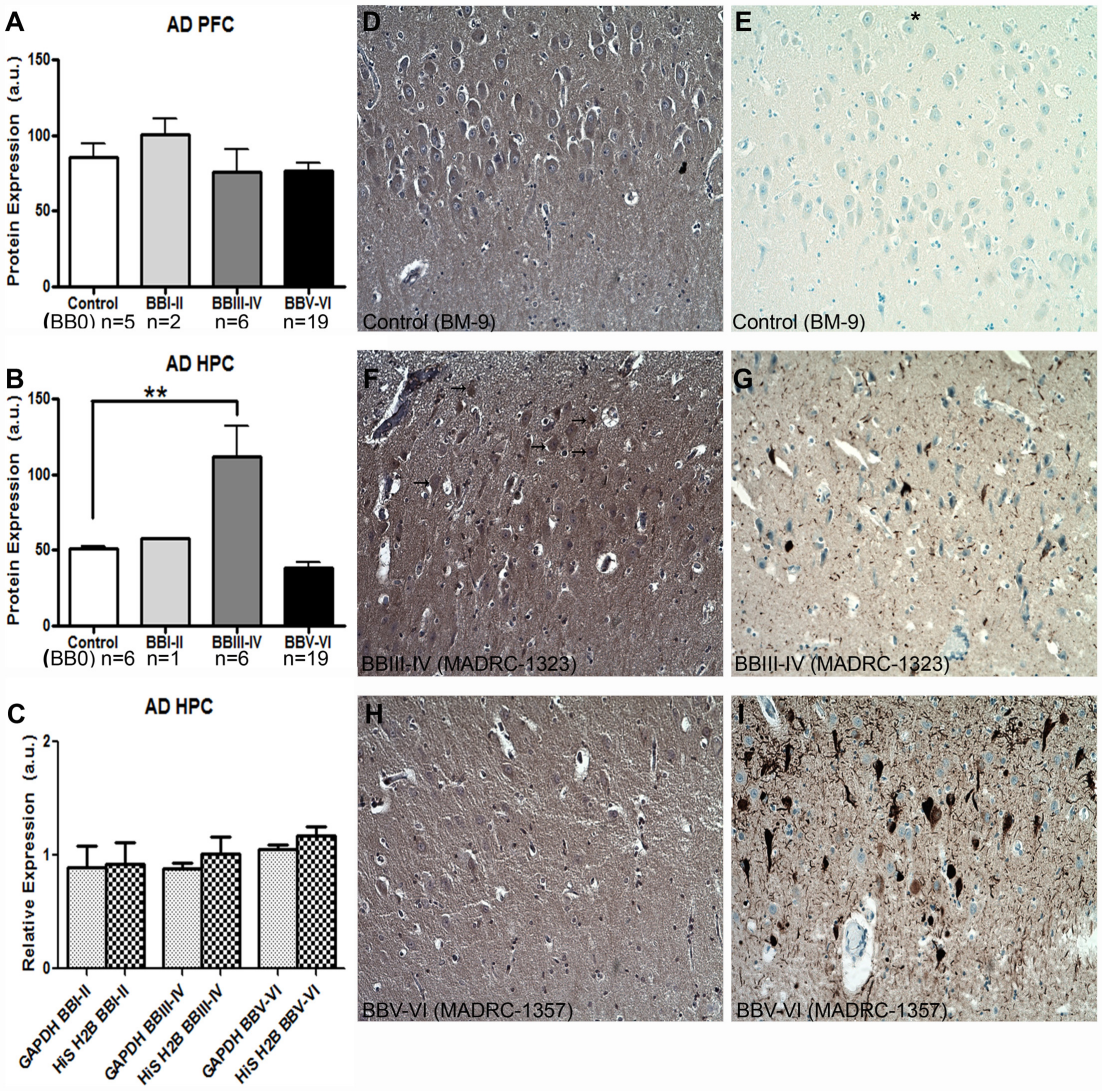
Capzb2 protein expression changes specifically in the hippocampal CA1 region (HPC) during progression of AD. (**A**) Capzb2 protein levels in PFC do not change significantly during the progressive stages of AD in comparison to control cases; (B) Capzb2 protein levels in HPC in AD BBIII-IV are significantly increased in comparison to control brains (**p<0.01, One-way ANOVA with subsequent Tukey's test); (C) when normalized to the expression in control brains, two unrelated proteins used as loading controls (GAPDH and histone H2b, His H2b) do not differ significantly in their level of expression in the hippocampal CA1 region regardless of the AD stage. Hippocampal sections of the representative cases form the progressive AD stages were processed for Capzb2 (**D,F,H**) and tau immunohistochemistry (**E,G,I**). As the AD progresses, neurofibrillary tangles highlighted by tau immunohistochemistry accumulate (**E,G,I**). and the density of CA1 pyramidal neurons decreases (**D–I**). Capzb2 signal is seen in the cytoplasms and neuropil, and appears stronger in AD BBIII-IV stage (arrows, **F**) in comparison to both normal control (BB 0; **D**) and advanced disease (AD BBV-VI; **H**). The asterisk in E indicates the direction of pial surface in **D–I**.

We examined Capzb2 protein expression levels in the whole tissue homogenates of hippocampal CA1 region and prefrontal cortex (Brodmann area 9) from the brains deposited at the Massachusetts Alzheimer Disease Research Center (MADRC) in the Massachusetts General Hospital and from brain autopsies performed at Boston Medical Center (BM) (Table 1). While Capzb2 expression remained steady in the prefrontal cortex (PFC) throughout progressive AD stages, it significantly increased in the hippocampal CA1 region (HPC) in BBIII-IV (p<0.01; One-way ANOVA, Figure 1A and 1B). To confirm the specificity of this increase, we examined, in addition to GAPDH (our loading control, see *Western Blot* section in MATERIALS AND METHODS), the expression of another unrelated protein, histone H2b (His H2b) (Figure 1C). The expression of GAPDH and His H2b in the examined hippocampi in AD BBI-VI did not change significantly across stages. We further examined the Capzb2 immunoreactivity in CA1 region in control, AD BBIII-IV and AD BBV-VI cases (Figure 1D–I). As expected[9], Capzb2 immunoreactivity was seen in cytoplasm and neuropil, and appeared most robust in the cytoplasm of pyramidal neurons in AD BBIII-IV cases (Figure 1F, arrows).

**Table 1:** Analyzed human autopsy brains.

Case number	Diagnosis	Age (yr)	Sex	Postmortem interval (hr)	RNA Integrity Number (RIN)
BM-1	Normal	58	M	19	
BM-6	Normal	65	M	4	
BM-9	Normal	72	F	23	
BM-16#	Normal	73	F	26	4.5
BM-17#	Normal	77	F	47	4.5
MADRC-1314	Normal	56	M	36	
MADRC-1339	Normal	79	F	48	
MADRC-1359*	AD BB I	75	M	NA	
MADRC-1301	AD BB I-II	85	M	24	
MADRC-1389	AD BB III-IV	69	N/A	24	
MADRC-972	AD BB III-IV	81	M	11	
MADRC-1323	AD BB III-IV	94	M	24	6.4
MADRC-912	AD BB IV	103	F	5	6
MADRC-1403#	AD BB IV	88	F	24	5.8
MADRC-1515#	AD BB IV	84	M	24	
MADRC-1345	AD BB IV-V	91	F	10	
MADRC-1372	AD BB V	74	F	8	
MADRC-1377	AD BB V	78	M	24	
MADRC-971	AD BB V	81	M	11	
MADRC-1052	AD BB V-VI	103	F	5.5	
MADRC-1058	AD BB VI	105	F	24	
MADRC-1202	AD BB VI	90	F	42	
MADRC-1269	AD BB VI	91	F	22	
MADRC-1303	AD BB VI	73	F	24	
MADRC-1307	AD BB VI	82	F	24	
MADRC-1315	AD BB VI	64	F	9	
MADRC-1325	AD BB VI	80	F	30	
MADRC-1329	AD BB VI	56	F	24	
MADRC-1331	AD BB VI	81	F	24	
MADRC-1341	AD BB VI	85	F	18	
MADRC-1344	AD BB VI	69	M	5	
MADRC-1353	AD BB VI	52	F	23	
MADRC-1357	AD BB VI	87	M	6	
MADRC-1360	AD BB VI	88	F	31	
MADRC-1363	AD BB VI	77	M	12	

*only PFC tissue was available;

# only HPC tissue analyzed.

**Figure 2:**
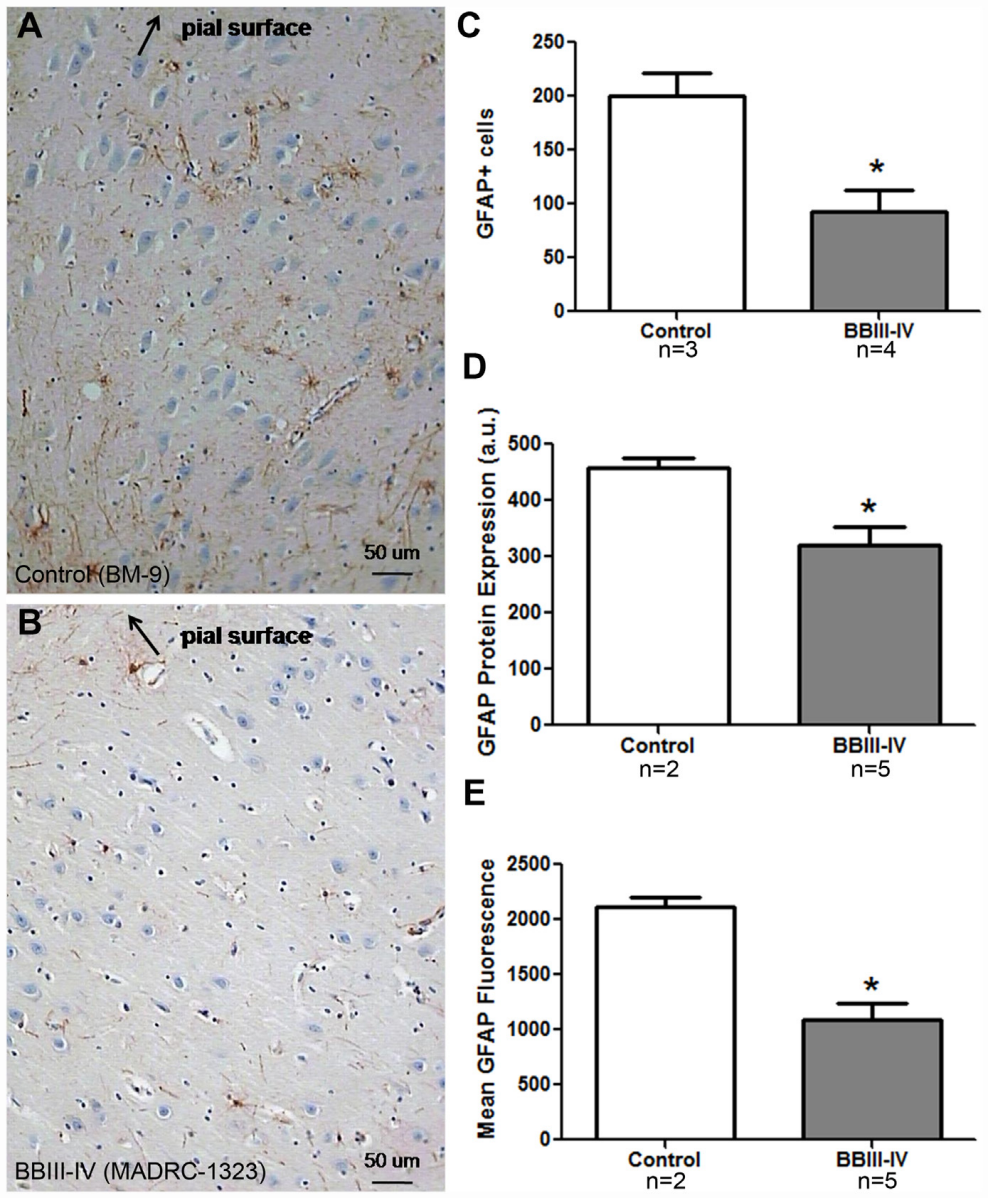
Reactive astrogliosis in CA1 region of AD BBIII-IV does not contribute to the increased Capzb2 expression. The number of GFAP positive cells (**A**–**C**; bar in **A**, **B**  = 50 µm), as well as the analysis of GFAP signal by Western Blot (**D**) and by Luminex capture sandwich immunoassay (**E**) indicate more reactive astrogliosis in control than in AD BB III-IV brains.

Since Capzb2 expression is not neuron-specific, we considered the possibility that the increase in Capzb2 signal might be due to reactive gliosis. We compared the number of GFAP- immunoreactive astrocytes in CA1 region of BBIII-IV AD and control brains (Figure 2A, 2B, and 2C). We also examined the GFAP expression by Western Blot (Figure 2D) and by Luminex analysis using the capture sandwich immunoassay (Figure 2E). Our results suggest that the observed increase in Capzb2 expression in the CA1 region of AD BBIII-IV brains could not be attributed to an increase in GFAP-immunoreactive glial cells.

### The increase in Capzb2 mRNA expression in CA1 pyramidal neurons in AD BBIII-IV is accompanied by the up-regulation of TrkB mRNA

**Figure 3:**
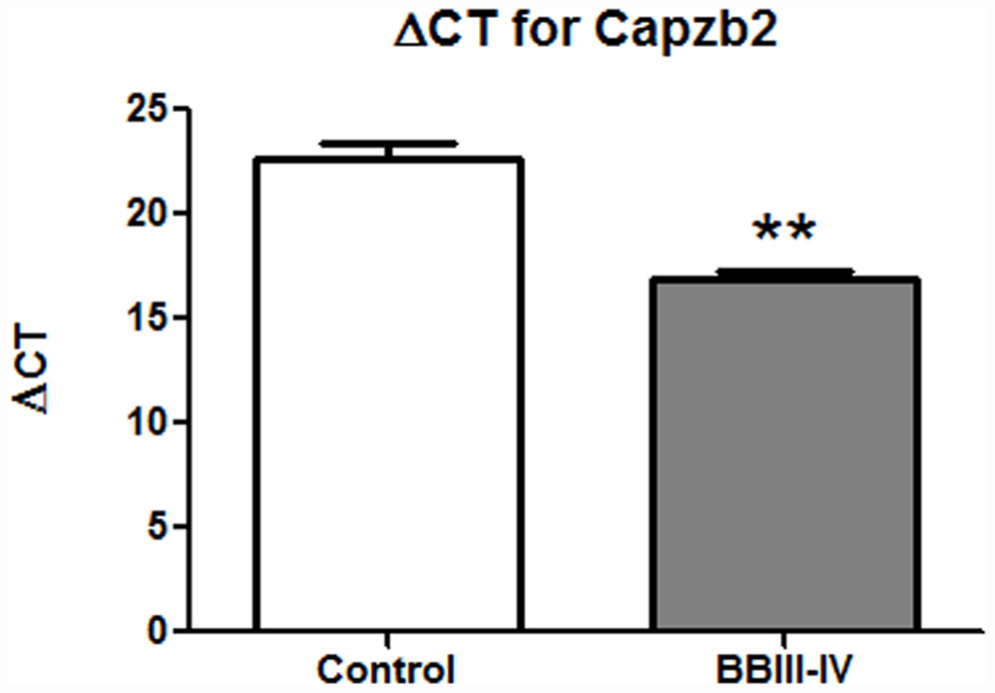
Capzb2 mRNA expression in CA1 pyramidal neurons of AD BBIII-IV is increased in comparison to control. Control brains (BM-16 and BM-17) cDNA shows higher ΔCT for Capzb2 gene than for the average of two AD BBIII-IV brains (MADRC-912 and MADRC-1323); **p<0.01, Student's t test.

In order to further elucidate the origin of the Capzb2 expression increase, we used laser capture microdissection (LCM) to examine a cell population particularly vulnerable to AD from the selected cases studied above with protein expression analysis. Visualization of hippocampal neurons prior to LCM was achieved using HistoGene stain (Figure S1). To generate cDNA for qPCR analysis, we obtained RNA from approximately 2, 000 CA1 pyramidal neurons from each of AD BBIII-IV (MADRC-912 and MADRC-1323) and control brains (BM-16 and BM-17) that had satisfactory RNA integrity numbers (RINs; Table 1, see also *RNA quality control* section in MATERIALS AND METHODS). The PCR quantification curves for control, house keeping gene 18S rRNA (Figures S2A, S3A, S3C) and for Capzb2 (Figure S2, S3B, S3D) were generated from each cDNA. Change in Cycle Threshold (ΔCT) between the Capzb2 and the house keeping gene 18S rRNA was calculated[23]. Two cDNA concentrations (full strength and 1∶2 dilution) were used to calculate ΔCT in order to confirm that the obtained ΔCT values were consistent with the cDNA concentrations. As duplicate samples were run, the average CT value for Capzb2 was subtracted from the average CT value for 18S rRNA, giving the ΔCT-Capzb2 value. The control brains' cDNAs show higher ΔCT for Capzb2 than cDNAs from AD BBIII-IV brains, indicating lower expression of Capzb2 in the control brains (Figure 3).

**Figure 4:**
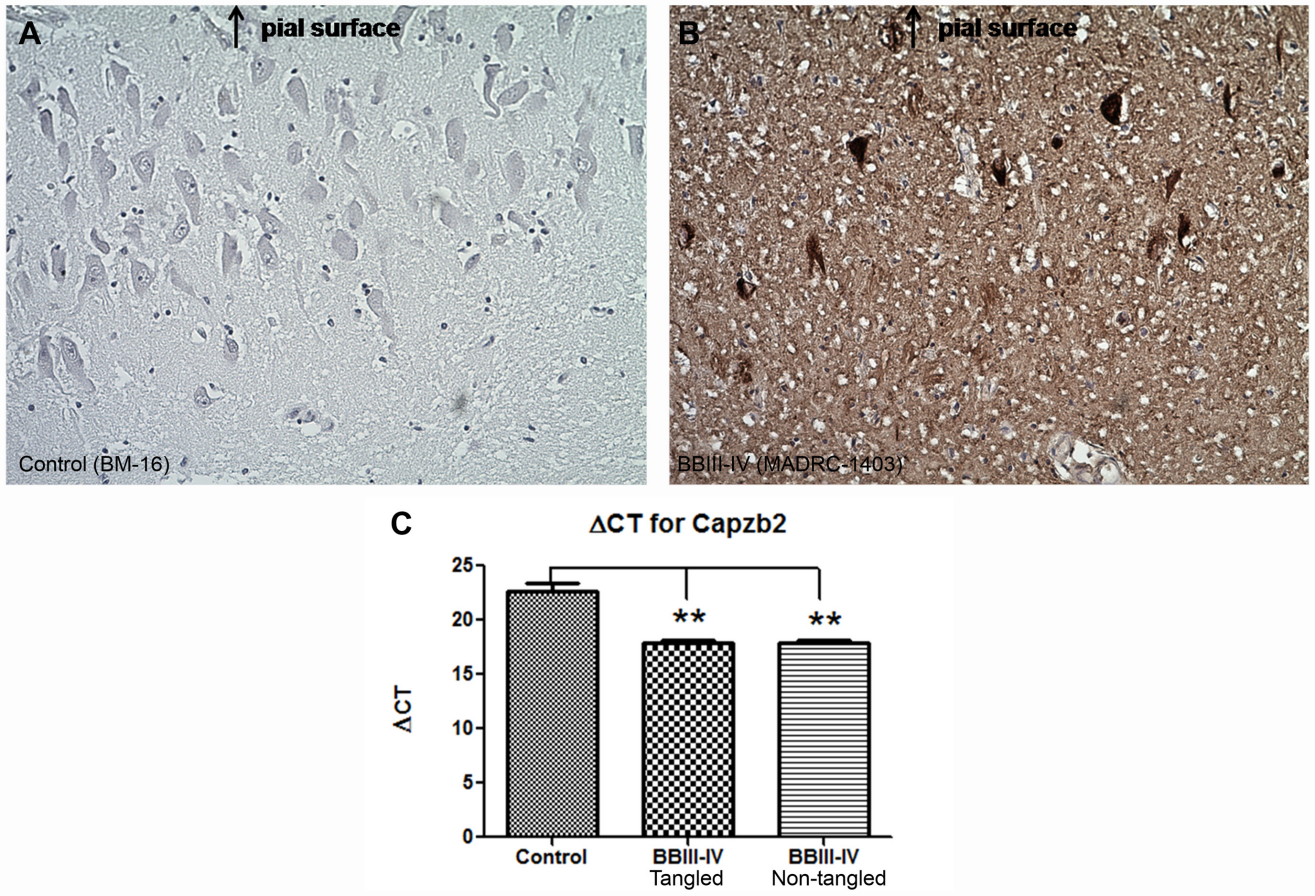
Capzb2 mRNA expression in CA1 pyramidal neurons is increased in AD BBIII-IV regardless of NFTs presence. NFTs are absent from the population of pyramidal neurons dissected from the control CA1 region (**A**), whereas moderate number of tangles are found in the pyramidal neurons dissected from AD BBIII-IV CA1 region (**B**; both tangled and non-tangled neurons as well as numerous dystrophic neurites are present). (**C**) cDNA of control CA1 pyramidal (non-tangled) neurons shows higher ΔCT value than the cDNAs from either tangled or non-tangled CA1 pyramidal neurons from AD BBIII-IV brain (**p<0.01; One-way ANOVA with subsequent Tukey's test); tangled and non-tangled CA1 pyramidal neurons at AD BBIII-IV have similar ΔCT values.

Since the subset of CA1 pyramidal neurons in AD BB III-IV displays defining AD intraneuronal cytoskeletal abnormalities, neurofibrillary tangles (NFTs), we next examined Capzb2 mRNA expression in relation to the presence of NFTs (Figure 4). Approximately 1,500 tangled and 1,500 non-tangled CA1 pyramidal neurons from AD BBIII-IV (MADRC-1403, Table 1) and 1,500 (non-tangled) CA1 pyramidal neurons from control brains (BM16 and BM-17, Table 1) were dissected for qPCR analysis (Figures 4 and S4). The mRNA levels of Capzb2 were higher in both tangled and non-tangled neurons of the AD BBIII-IV brain compared to the controls, while similar in tangled and non-tangled neurons of the AD BBIII-IV brain (Figure 4C).

**Figure 5:**
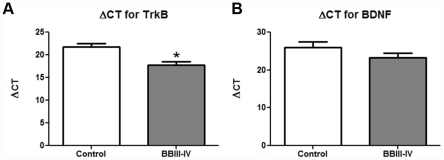
The expression of TrkB mRNAs in CA1 pyramidal neurons of AD BBIII-IV is increased in comparison to control. The cDNA from two control brains (BB0; BM-16 and BM-17) CA1 pyramidal neurons has higher ΔCT value for TrkB (**A**) in comparison to the average of two AD BBIII-IV (MADRC-912 and MADRC-1323) values indicating lower amount of TrkB mRNA; *p<0.05, Student's t test. BDNF mRNA levels in the examined AD BB III-IV cases are not significantly different from those found in control cases (**B**).

Because of their role in memory and cognition, BDNF and TrkB have been previously studied in the brains of AD patients[24]–[27], but without the information on Braak and Braak stage of AD associated neuropathology. Braak and Braak staging, introduced in 1997 and widely accepted thereafter, is based on the extent of cortical involvement by neurofibrillary tangles[28]. Using LCM and qPCR, we examined the expression of BDNF and TrkB mRNAs in the developing, mid-stage (BBIII-IV), rather than in the end-stage (BBVI) AD[24] (see DISCUSSION). Similar to Capzb2, the mRNA level of TrkB in the CA1 neurons in AD BBIII-IV brains was higher than that in the control brains (Figures 5 and S5).

## Discussion

We previously demonstrated that RNAi-mediated silencing of Capzb2 in cultured hippocampal neurons resulted in short, dystrophic neurites[9] reminiscent of the cytoskeletal changes associated with neurodegeneration in AD. Cytoskeletal abnormalities that included dystrophic neurites, decreased dendritic areas, and decreased spine numbers have been described in hippocampal neurons of mice that carried an APP transgene with multiple mutations associated with familial AD[29]. Whereas at 3 months of age APP transgenic hippocampal neurons showed no cytoskeletal abnormalities, at 11 months of age evidence of degeneration existed in all the parameters examined. Many neurons displayed dystrophic neurites, reduced dendritic arborization, and loss of spines[29]. Most intriguing, however, were data suggestive of regenerative activity at a time-point in between - at 8 months of age, as rare dystrophic neurites became noticeable, a small *increase* in the number of spines and total dendritic area was observed in APP transgenic neurons in comparison to controls[29]. Dendritic proliferation and sprouting in human AD brain have been observed by Golgi impregnation[30] and by MAP2 and tau immunohistochemistry highlighting growth cones present both in perisomatic dendrites and in distal, dystrophic neurites[20]. Thus, an AD transgenic model and a human diseased brain share the hallmark of cytoskeletal reorganization underlying degenerative (dystrophic neurites) as well as regenerative changes (sprouting of processes and the presence of growth cones).

Here we report an increase in the expression of Capzb2, a protein necessary for normal growth cone morphology and neurite length[9], specifically in the hippocampal CA1 region at mid-stage AD (BBIII-IV) (Figure 1). This increase in Capzb2 expression could not have been aided by the astrocytic gliosis was less prominent in AD BBIII-IV brains than in control brains (Figure 2). Consistent with our findings are previously reported GFAP mRNA age-related increase in archi- and neo-cortex of both rats and humans[31]and widespread astrocytic gliosis in both Alzheimer's and normal aging cerebrum[32]. Important for our analysis was the definition of normal, “control” cases considering that the cognitive function appears to be degraded in the non-demented individuals who do exhibit AD- related neuropathological changes[33]. We performed tau immunohistochemistry on all of the studied hippocampal blocks and considered as controls only those without any neurofibrillary tangles (BB 0; Figures 1, 2, and data not shown).

The protein expression analysis of the CA1 region is supported by the data on Capzb2 mRNA expression in hippocampal CA1 neurons obtained via LCM (Figure 3). Several pathologic features of AD make cell population expression profiling a rational alternative to whole brain homogenates: 1) defined vulnerable neuronal populations; 2) distinct intracellular abnormalities found in some but not all of the cells; and 3) the poor understanding of the relationship between the presence of distinct extra- and intra- cellular abnormalities and neuronal function and survival. The importance of cell population expression profiles is in their potential to identify new rational targets for pharmacotherapeutic interventions in progressive neurodegenerative disorders such as AD. Mature brain derived neurotrophic factor or BDNF shows high-affinity binding to the neurotrophic tyrosine kinase receptor TrkB. The molecular basis for new learning is a rapid effect on synaptic transmission (short-term memory) followed by persistent changes, such as long-term potentiation, across neuronal synapses (synaptic plasticity) that consolidate long-term memories. BDNF appears to facilitate both synaptic transmission and long-term potentiation in glutamergic hippocampal synapses acting through post-synaptic TrkB receptors[34]. Conditional knock-out mice lacking forebrain TrkB and mice treated with blocking antibodies to hippocampal BDNF[35] show decreased development of LTP and impaired learning. Animal studies suggest that as β-amyloid accumulates in the hippocampus, it interferes with the normal function of BDNF[36]. One clinical study suggested that patients with AD have low whereas those with mild cognitive impairment (a stage preceding clinical dementia) had intermediate BDNF levels compared to normal controls[37]; others have suggested raised BDNF levels in early AD[38], [39]. A well-documented role of BDNF/TrkB signaling in memory formation and retention via cytoskeletal reorganization[16], [17] has been driving the interest in studying the expression of these molecules in relation to AD pathological hallmarks[24]. Using immunohistochemical double-labeling, Fujimura et al. (2010) showed that the number of tangled neurons that expressed BDNF was similar in AD and controls[24]. In the same study, quantitative RT-PCR analysis of AD and control hippocampal neurons, revealed that the expressions of BDNF and TrkB in AD were equally low in Aβ-containing and Aβ-free neurons and lower than that in control brains[24]. Importantly, the three AD cases examined were all in advanced BB stages (VI, VI, and V). Considering that a fully developed neurodegenerative process may obscure the mechanisms driving the disease progression, we examined the expression of BDNF and TrkB mRNAs in mid-stage AD (BBIII-IV) when Capzb2 was specifically up-regulated (Figure 1, 2, 3, and 4). Similar to Capzb2, the expression of TrkB in hippocampal CA1 neurons in AD BBIII-IV hippocampi was higher than that in the control (Figure 5). Importantly, our control hippocampi did not contain neurofibrillary tangles or dystrophic neurites (Figure 4A) whereas the three cases used as controls by Fujimura at al. (2010) did demonstrate AD-related changes: two brains were categorized as BBI and one as BBII[24].

Understanding the role of BDNF/TrkB signaling in AD pathogenesis could be of great clinical and public health importance since BDNF is inducible and may be one of the key molecules mediating the beneficial effect of certain lifestyle measures (lower caloric intake, increased aerobic physical activity, environmental enrichment)[40]–[42]on the risk of developing dementia. These modifiable environmental interventions increase brain and serum BDNF levels[43]–[46]. Aerobic exercise for example induces both short-term and longer-term increases in circulating BDNF levels, in parallel with improvements in memory performance[47].

Our study shows that the expression of TrkB and Capzb2 mRNAs in hippocampal CA1 pyramidal neurons during mid-stage AD (BBIII-IV) is up-regulated in comparison to the control hippocampus containing no AD- related cytoskeletal abnormalities (Figure 3, 4, and 5). Similar levels of Capzb2 mRNA expression in tangled and non-tangled neurons in mid-stage AD (Figure 4) support the observations that both populations undergo cytoskeletal reorganization that includes regenerative changes[20], [30]. It is conceivable that the progression of AD and concomitant cytoskeletal reorganization is accompanied by the expression of molecular markers of endogenous neuronal resistance to degeneration and/or molecular markers of regeneration. The expression of these markers may reflect neuronal changes associated with regeneration and possibly memory performance warranting future correlation studies with the cognitive status information in individual AD patients.

## Methods

### Ethics Statement

The Institutional Review Board at Boston University School of Medicine provided an exemption for the protocol describing research on postmortem human brain tissue analyzed in this study.

We examined the hippocampal CA1 region (HPC) and the prefrontal cortex (PFC, Brodmann area 9) available from the brains deposited at Massachusetts Alzheimer Disease Research Center (MADRC) at Massachusetts General Hospital and from brain autopsies performed at Boston Medical Center (BM), (Table 1).

### Immunohistochemistry

Hippocampal blocks (level of lateral geniculate nucleus) were fixed in 4% paraformaldehyde and embedded in paraffin. For detection of Capzb2 signal in hippocampal CA1 neurons, 10-µm sections were incubated with an affinity isolated anti-CAPZB antibody produced in rabbit (designed for immunohistochemistry application, Sigma-Aldrich, 1∶100) for 30 min at RT, washed in phosphate buffered saline (pH 7.4; PBS), incubated with a secondary antibody (Universal HRP multimer containing cocktail of HRP labeled goat anti-rabbit) and visualized by DAB (Figure 1D, 1F, 1H). For the detection of reactive astrocytes, sections were incubated with a rabbit polyclonal anti-GFAP antibody (1: 2,000, Dako) overnight at 4°C, washed in PBS, incubated with a secondary antibody (Universal HRP multimer containing cocktail of HRP labeled goat anti-rabbit) and visualized by DAB (Figure 2A and 2B). Tiled images (470 µm ×470 µm) were acquired from the CA1 region under the 20x magnification. GFAP- immunoreactive astrocytes were counted in 20 tiled images from the CA1 region (a total area of 4.42 mm^2^).

For the detection of neurofibrillary tangles (Figure 1E, 1G, 1I, 4A and 4B), paraffin sections were incubated with anti-tau antibody (rabbit polyclonal, 1: 5,000, Dako, or mouse monoclonal, PHF-1, 1∶1,000; a gift from Dr. Peter Davies) overnight at 4°C, washed with PBS, incubated with a secondary antibody incubation (Super Sensitive Link Label IHC Detection Systems kit, Biogenex) and visualized by DAB. Slides were counterstained with hematoxylin and dehydrated with ethanol and xylene. Before laser capturing to obtain neurons for qPCR analysis in Figure 4, 10-µm frozen sections were incubated with the same anti-tau antibody (PHF-1, 1∶50) for 10 min on ice, washed with PBS, incubated with anti-mouse IgG HRP (Pierce, 1∶50) for 10 min and visualized by DAB. Slides were counterstained with hematoxylin and dehydrated with ethanol and xylene.

### Western Blot

The whole tissue lysates of hippocampal CA1 regions were prepared in RIPA buffer containing protease inhibitors (Roche). The final protein concentration of the lysates were determined by BCA assay. Lysates were subjected to SDS-PAGE and immunoblotting with anti-Capzb2 (DSHB, 1: 1,000), anti-GFAP (Sigma, 1: 1,500), anti-histone H2B (Abcam, 1: 2,000), and anti-GAPDH (Ambion, 1∶10,000) using appropriate secondary HRP-conjugated antibodies at 1∶5,000 (Santa Cruz) (6 repetitions). A chemiluminescent detection system (Pierce) was used to visualize protein expression on an electronic capture imager (Kodak). Densitometry measurements were performed using ImageJ version 1.37v software (National Institutes of Health). For Capzb2 expression, values were calculated as (intensity x area of Capzb2 signal)/(intensity x area of GAPDH signal) (Figure 1A and 1B). In Figure 1C the expressions (intensity x area of signal) of two unrelated proteins (GAPDH and Histone H2b) in each BB group were normalized to their expressions in the control group.

### Luminex analysis

A mouse monocolonal GFAP antibody (GFAP mAb, 25 µg, Sigma) was coupled to 1.25 million Microplex microspheres 133 (Luminex) using EDC (Thermo Scientific) and Sulfo-NHS (Thermo Scientific) as directed by the manufacturer protocol. A rabbit polyclonal GFAP antibody (GFAP pAb, 145 µg, Dako) was coupled to biotin using EZ-link Sulfo-NHS-LC-Biotin reagent (Pierce) as directed by the manufacturer protocol. Brain lysates (0.5 µg/50 µl) were incubated with 50 µl of GFAP mAb-microspheres (100 MS/µl) in a 100 µl reaction for 30 min at room temperature (RT) in a 1.2 µm filter plate (Millipore) with agitation. The GFAP mAb-microspheres were washed twice with PBS-1%BSA and resuspended in 50 µl PBS-1% BSA. 50 ul of GFAP pAb-biotin (50 µl, 1 µg/ml) was then added and the mixture was incubated at RT for 30 min. Microspheres were then washed twice, resuspended in 50 µl PBS-1% BSA and 50 µl of Streptavidin-R-phycoerythrin reporter (Invitrogen) (4 µg/ml), and incubated for 30 min at RT. Upon another wash, the microsphere complexes were resuspended in 100 µl of PBS-1% BSA and analyzed on a Luminex 200. GFAP expression was reported as the mean fluorescence signal per 100 microspheres. The assay was performed three times.

### RNA quality control

We have examined the RNA quality of the hippocampal blocks used for LCM and subsequent qPCR analysis by the commonly accepted quantitative digital analysis of Agilent 2100 Bioanalyzer generated electropherograms. The resulting RNA Integrity Number (RIN) of 1 (poor) to 10 (best) is obtained based on the presence and ratio of the 5S, 18S and 28S ribosomal RNA peaks in a sample of total cellular RNA. Banked postmortem brain tissues across institutions have been reported to display range of RIN values from 2.9 to 9.2 (mean  = 6.8+/− 1.0)[48]. We have found that even partially degraded RNA with a RIN value of 3.1 shows fairly well preserved mRNA profile (unpublished data). The hippocampi used for qPCR displayed RIN values from 4.5 to 6.4 (Table 1).

### Laser Capture Microdissection (LCM)

In preparation for LCM, frozen blocks of the hippocampal tissue (level of the lateral geniculate) were oriented and affixed to cryotome chucks with OCT embedding compound. Ten µm cryosections were stained to visualize cells (Figure S1). We found that approximately 2,000 CA1 pyramidal neurons yielded enough cDNA for qPCR of four genes (our genes of interest - Capzb2, BDNF, TrkB - and housekeeping gene, 18S rRNA). Veritas LCM system was used to collect CA1 pyramidal neurons onto optically clear collecting caps that contain a thin plastic film impregnated with a laser sensitive dye. Caps were placed over the area of interest and illuminated with a monochromatic IR laser pulse which selectively heats the plastic/dye combination at a set size (sizes are set anywhere between a diameter of 4 and 50 µm) and lifts the precisely microdissected underlying tissue when the cap is lifted. The isolated material was viewed to verify its integrity before RNA extraction. For the visualization of neurons, each section was lightly fixed in 70% ethanol (EtOH), rinsed with RNase-free dH2O, incubated in HistoGene stain solution (MDS Analytical Technologies), and dehydrated in ethanol solutions and xylene. For the visualization of neurofibrillary tangles, see *Immunohistochemistry* section. After neurons were collected, RNA was prepared using the Arcturus picopure RNA isolation system (Arcturus, MDS Analytical Technologies). Briefly, plastic LCM collecting caps with captured neurons were incubated at 42°C for 30 minutes in 25 µl of GITC-containing extraction buffer. After brief centrifugation, tubes containing the extraction buffer were frozen at -80°C. Samples are thawed and pooled prior to purification on Picopure RNA columns. The RNAs from the cell homogenate were attached to the column filter. After DNase treatment, genomic DNA was removed. Total cellular RNA from each column was eluted in 12 µl of elution buffer. RNA was converted into cDNAs using a 20 µl total reaction volume via the Standard Superscript III (Invitrogen) protocol for qPCR analysis.

### Primer design and qPCR analysis

For Capzb2 PCR, multiple pairs of primers were generated by the Primer3 software and produced by Invitrogen. The PCR products obtained with these primers were tested in Bioanalzyer gel and Bioanalzyer Electropherograms (Figure S2). The successful Capzb2 primers (forward: gcaaatcgagaaaaacctca; reverse: ctccaagggagggtcatact) were used in qPCR analysis. Similarly, successful primers for BDNF (forward: cagctgccttgatggttact; reverse: ccaatgatgtcaagcctctt) and TrkB (forward: ggacgtgtacagcactgact; reverse: tgccataggtgaaaatctcc) were obtained. All the primers were tested using standard cDNA from normal human brain as template provided by ATRC Reagent Bank. The Bank also provided primers for PCR of 18S rRNA, a house keeping gene often used as an internal control.

### Statistical analysis

One way ANOVA analysis was performed in multiple groups comparisons (Figure 1 and 4C) except for the comparison between the control group and BB stage I-II in Figure 1B where Student's t test was performed because of only one hippocampus specimen (Table 1). Student's t test was also performed in the rest of the statistical analysis where two groups/samples were compared (Figure 2C, 2D, 2E, 3, 4C, and 5).

## Supporting Information

Figure S1 Hippocampal CA1 region prepared for LCM of pyramidal neurons. Visualization of the cells using HistoGene stain at 2x (A: area magnified from the red square on the insert) and 20x (B: pyramidal neurons are identified for capturing by LCM).
(3.54 MB TIF)

Figure S2 Bioanalyzer gel of PCR products obtained with primers for Capzb2 and 18S rRNA genes (A) and electropherogram for Capzb2 PCR product (B). The bioanalyzer gel indicates the correct sizes of PCR products: Capzb2 -198 bp, and 18S rRNA -177 bp. The electropherogram shows the number of fluorescent units (FU) on the Y axis that corresponds with each bp size on the X axis. The highest peak is at 198 bp (the correct size of Capzb2 PCR product).
(1.45 MB TIF)

Figure S3 qPCRs for 18S rRNA (A, C) and Capzb2 (B, D). These figures complement Figure 3. The Y axis shows the amplification and the X axis shows the number of cycles. Each line traces the amplification of either full strength or 1∶2 diluted cDNA from analyzed cases: BM-16 (control, BB0), BM-17 (control, BB0), MADRC-912 (AD BBIII-IV), and MADRC-1323 (AD BBIII-IV). Reactions with dilutions of each cDNA were run in duplicates. qPCRs for 18S rRNA for control cases (A) show control case BM-16 in navy (full strength) and blue (1∶2 dilution) and control case BM-17 in dark green (full strength) and light green (1∶2 dilution). The same shades are used for Capzb2 qPCRs in those cases (B). qPCRs in AD BBIII-IV cases for 18S rRNA (C) and for Capzb2 (D) show AD BBIV case MADRC-912 in navy (full strength) and blue (1∶2 dilution); AD BBIII-IV case MADRC-1323 is shown in dark green (full strength) and light green (1∶2 dilution).
(1.67 MB TIF)

Figure S4 qPCRs for 18S rRNA (A, B) and Capzb2 (C, D). These figures complement Figure 4. Each line traces the amplification of either full strength or 1∶2 diluted cDNA (shades of the same color; in duplicates) in non-tangled neurons from control (BB0) cases BM-16 (full strength -navy; 1∶2 dilution -blue) and BM-17 (full strength -dark green; 1∶2 dilution -light green) for 18S rRNA (A) and Capzb2 (C). qPCRs for 18S rRNA in tangled neurons from AD BBIV case MADRC-1403 is in navy (full strength) and blue (1∶2 dilution) and in non-tangled neurons from the same section in dark green (full strength) and light green (1∶2 dilution) (B). qPCRs for Capzb2 in tangled (full strength -navy; 1∶2 dilution -blue) and non-tangled (full strength -dark green; 1∶2 dilution -light green) neurons of AD BBIV case MADRC-1403 (D).
(1.71 MB TIF)

Figure S5 qPCRs for 18S rRNA in control cases (A, E) and in AD BBIII-IV cases (C, G); BDNF in control (B) and AD BBIII-IV (D) cases; TrkB in control (F) and AD BBIII-IV (H) cases. These figures complement Figure 5. Each line traces the amplification of either full strength or 1∶2 diluted of analyzed control cases (BM-16 and BM-17) and AD BBIII-IV cases (MADRC-912 and MADRC-1323). Reactions with dilutions of each cDNA were run in duplicates. Navy (full strength) and blue (1∶2 dilution) labelings are used for amplicons from control case BM-16 (A, B, E, F) and from AD BBIV case MADRC-912 (C, D, G, H). Shades of green are amplicons from control case BM-17 (A, B, E, F) and from AD BBIV case MADRC-1323 (C, D, G, H) (full strength -dark green; 1∶2 dilution -light green).
(3.43 MB TIF)
