# Supplementary figures and images for: Modulators of Cytoskeletal Reorganization in CA1 Hippocampal Neurons Show Increased Expression in Patients at Mid-Stage Alzheimer's Disease
Source: PLoS One. 2010 Oct 13;5(10):e13337. doi: 10.1371/journal.pone.0013337 (PMC2954170; doi:10.1371/journal.pone.0013337)

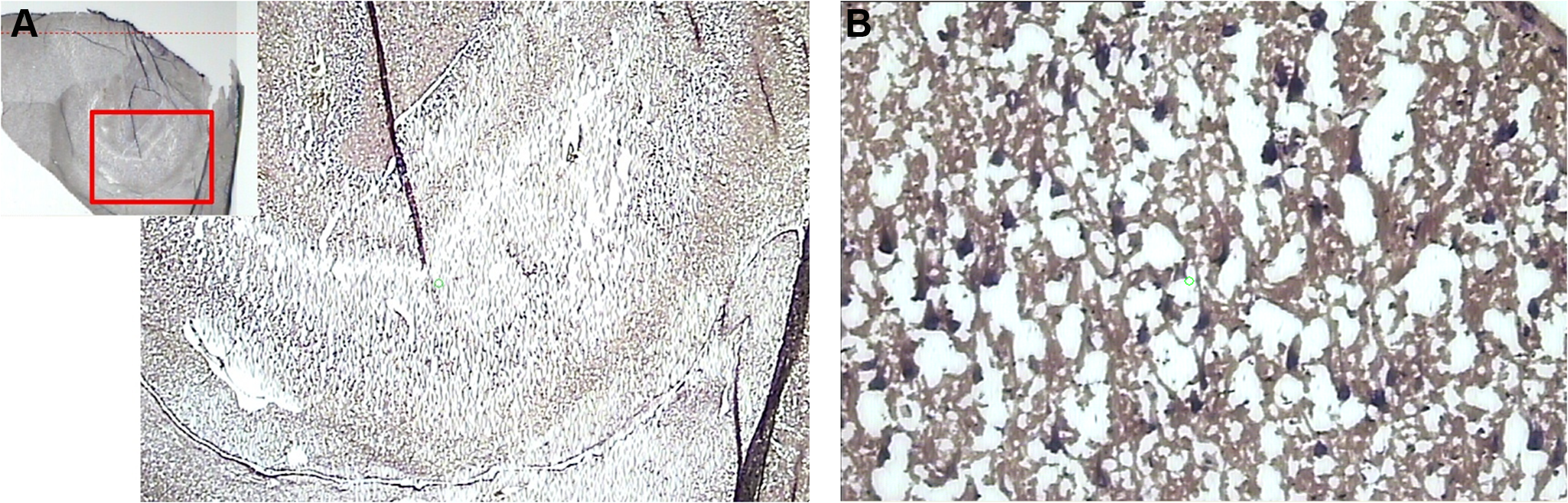

Supplement: Figure S1 — Hippocampal CA1 region prepared for LCM of pyramidal neurons. Visualization of the cells using HistoGene stain at 2x (A: area magnified from the red square on the insert) and 20x (B: pyramidal neurons are identified for capturing by LCM). (3.54 MB TIF) [file pone.0013337.s001.tif]

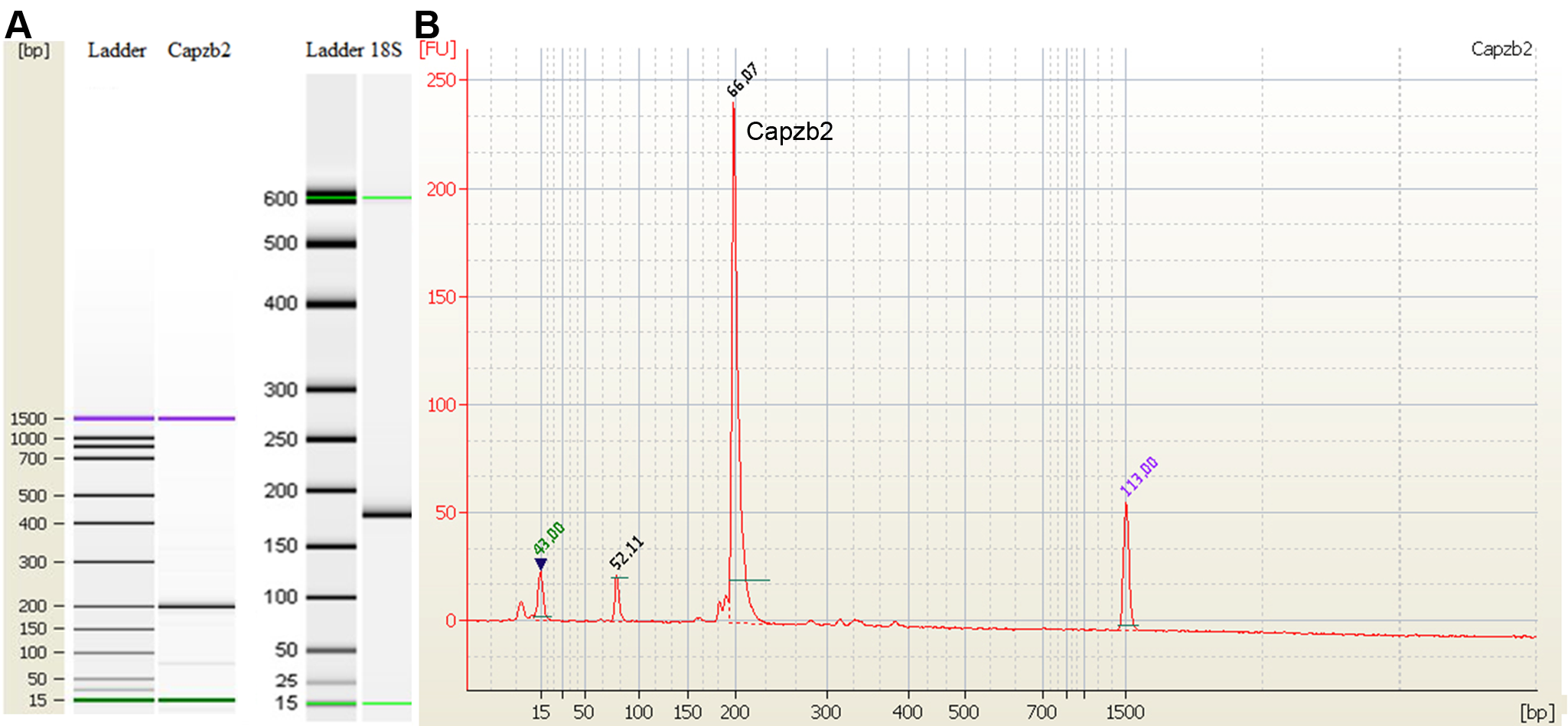

Supplement: Figure S2 — Bioanalyzer gel of PCR products obtained with primers for Capzb2 and 18S rRNA genes (A) and electropherogram for Capzb2 PCR product (B). The bioanalyzer gel indicates the correct sizes of PCR products: Capzb2 -198 bp, and 18S rRNA -177 bp. The electropherogram shows the number of fluorescent units (FU) on the Y axis that corresponds with each bp size on the X axis. The highest peak is at 198 bp (the correct size of Capzb2 PCR product). (1.45 MB TIF) [file pone.0013337.s002.tif]

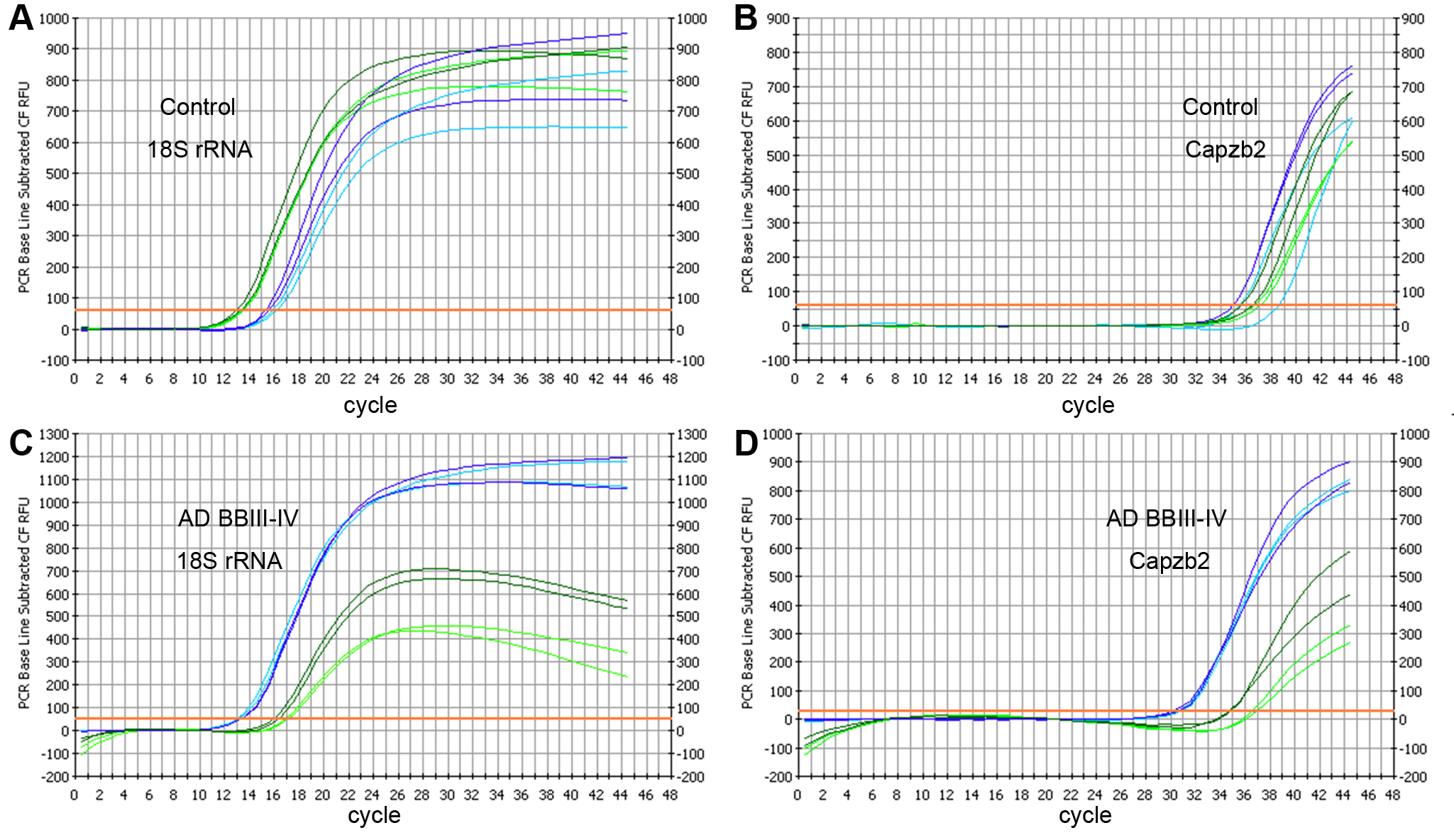

Supplement: Figure S3 — qPCRs for 18S rRNA (A, C) and Capzb2 (B, D). These figures complement Figure 3. The Y axis shows the amplification and the X axis shows the number of cycles. Each line traces the amplification of either full strength or 1∶2 diluted cDNA from analyzed cases: BM-16 (control, BB0), BM-17 (control, BB0), MADRC-912 (AD BBIII-IV), and MADRC-1323 (AD BBIII-IV). Reactions with dilutions of each cDNA were run in duplicates. qPCRs for 18S rRNA for control cases (A) show control case BM-16 in navy (full strength) and blue (1∶2 dilution) and control case BM-17 in dark green (full strength) and light green (1∶2 dilution). The same shades are used for Capzb2 qPCRs in those cases (B). qPCRs in AD BBIII-IV cases for 18S rRNA (C) and for Capzb2 (D) show AD BBIV case MADRC-912 in navy (full strength) and blue (1∶2 dilution); AD BBIII-IV case MADRC-1323 is shown in dark green (full strength) and light green (1∶2 dilution). (1.67 MB TIF) [file pone.0013337.s003.tif]

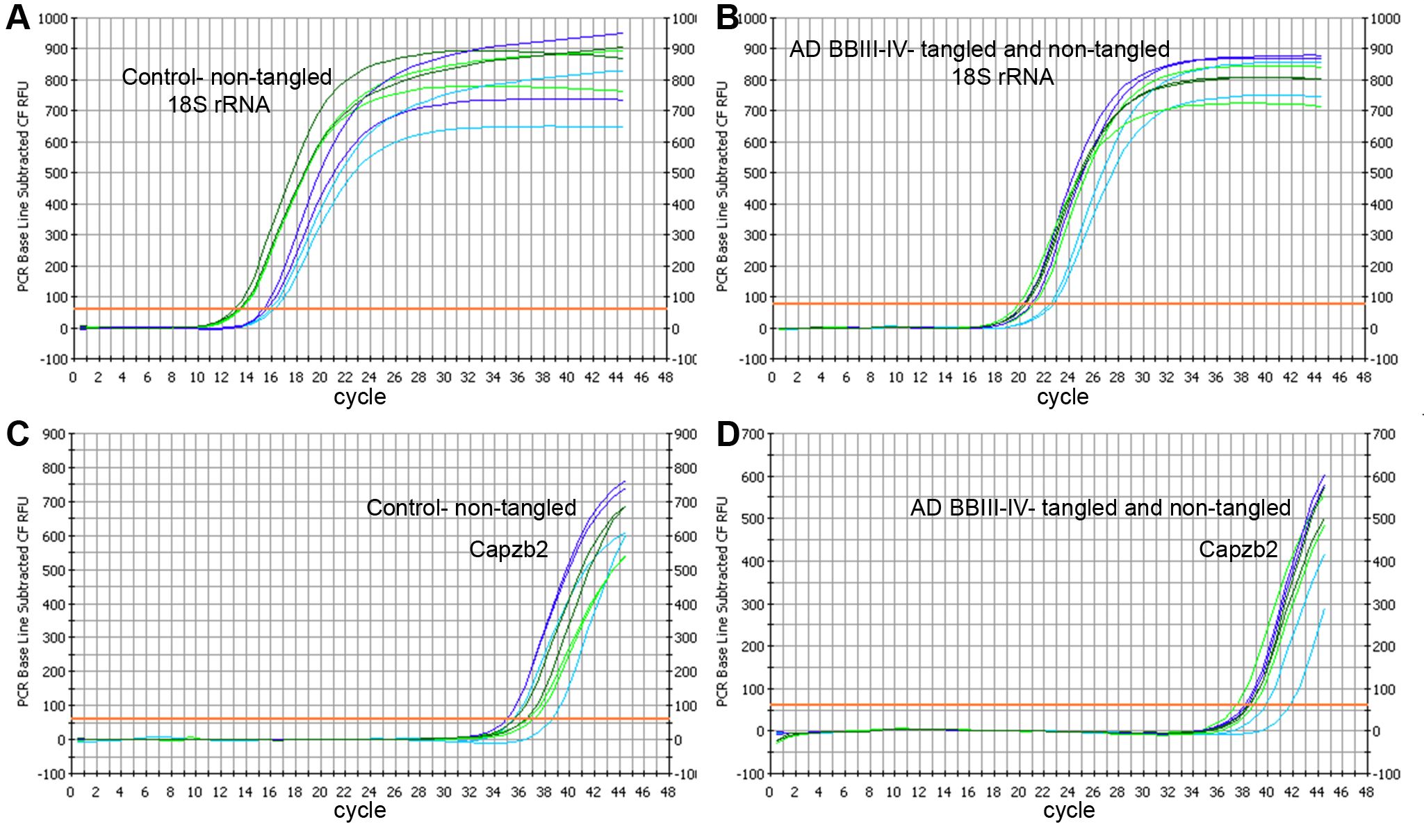

Supplement: Figure S4 — qPCRs for 18S rRNA (A, B) and Capzb2 (C, D). These figures complement Figure 4. Each line traces the amplification of either full strength or 1∶2 diluted cDNA (shades of the same color; in duplicates) in non-tangled neurons from control (BB0) cases BM-16 (full strength -navy; 1∶2 dilution -blue) and BM-17 (full strength -dark green; 1∶2 dilution -light green) for 18S rRNA (A) and Capzb2 (C). qPCRs for 18S rRNA in tangled neurons from AD BBIV case MADRC-1403 is in navy (full strength) and blue (1∶2 dilution) and in non-tangled neurons from the same section in dark green (full strength) and light green (1∶2 dilution) (B). qPCRs for Capzb2 in tangled (full strength -navy; 1∶2 dilution -blue) and non-tangled (full strength -dark green; 1∶2 dilution -light green) neurons of AD BBIV case MADRC-1403 (D). (1.71 MB TIF) [file pone.0013337.s004.tif]

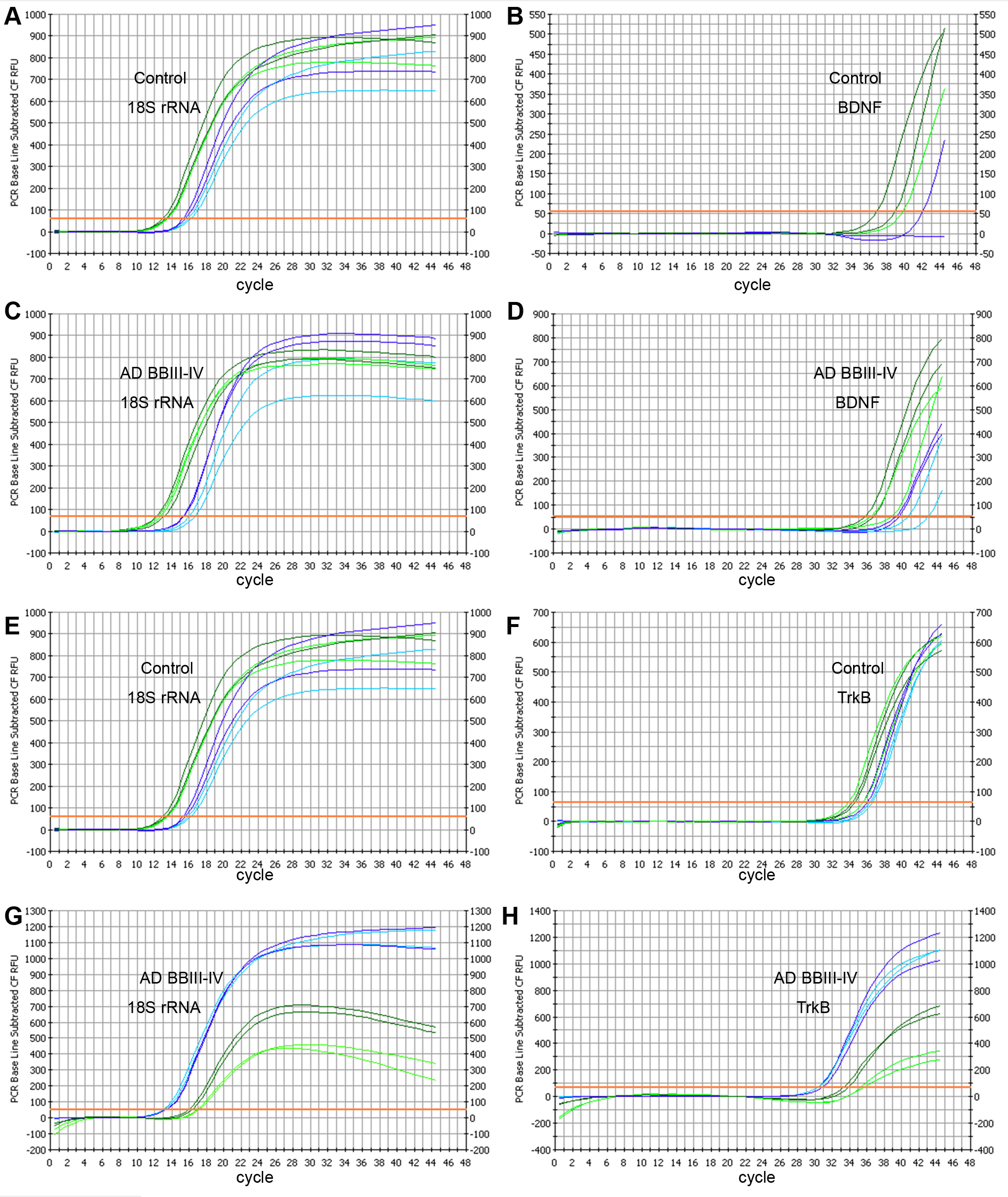

Supplement: Figure S5 — qPCRs for 18S rRNA in control cases (A, E) and in AD BBIII-IV cases (C, G); BDNF in control (B) and AD BBIII-IV (D) cases; TrkB in control (F) and AD BBIII-IV (H) cases. These figures complement Figure 5. Each line traces the amplification of either full strength or 1∶2 diluted of analyzed control cases (BM-16 and BM-17) and AD BBIII-IV cases (MADRC-912 and MADRC-1323). Reactions with dilutions of each cDNA were run in duplicates. Navy (full strength) and blue (1∶2 dilution) labelings are used for amplicons from control case BM-16 (A, B, E, F) and from AD BBIV case MADRC-912 (C, D, G, H). Shades of green are amplicons from control case BM-17 (A, B, E, F) and from AD BBIV case MADRC-1323 (C, D, G, H) (full strength -dark green; 1∶2 dilution -light green). (3.43 MB TIF) [file pone.0013337.s005.tif]
